# Dine in or take out dataset: user behavior in an interactive virtual reality café

**DOI:** 10.1016/j.dib.2026.113026

**Published:** 2026-06-24

**Authors:** Elza Ibragimov, Natasha Kholgade Banerjee, Sean Banerjee, Ashutosh Shivakumar

**Affiliations:** Wright State University, Department of Computer Science and Engineering, 3640 Colonel Glenn Highway, Dayton, OH, 45435, USA

**Keywords:** Social VR, Social phobia, Loneliness, Waiting in VR, Frustration, Anxiety

## Abstract

We present the Dine In or Take Out Dataset, a virtual reality dataset collected using a Meta Quest Pro, to understand how predominantly Gen Z individuals immersed in a virtual café opt to make the choice of dining in or taking food to go. In our dataset, participants completed four different treatments: no wait for maître d' with no wait for food, no wait for maître d' with 3-minute wait for food, 3-minute wait for maître d' with no wait for food, and 3-minute wait for maître d' with 3-minute wait for food, with treatment orders being randomized for each participant. Our dataset consists of 35 participants interacting with a virtual maître d' non-playable character (NPC) in a virtual cafe environment to order food for dine-in or take-out. Our virtual café was designed using Unity and consists of standing and seated sections found in a typical real-world café and includes four NPCs, the interacting maître d' and 3 seated patrons. To replicate the sounds of a typical café, we generate background sounds of clacking utensils and plates, pouring of liquid, distant conversations, ambient music along with the dialog between the NPC and the participant. Participants provided their demographic information and completed the standard Frustration Distraction Scale, Revised UCLA-20 Loneliness Scale, Social Phobia Inventory, State Trait Anxiety Inventory, which measure frustration and discomfort intolerance, loneliness, social phobia, and anxiety respectively, prior to immersion. After each treatment, participants retake the State Trait Anxiety Inventory to measure changes in anxiety and rate the proximity, noise level, frustration due to delays, frustration due to a sense of isolation, frustration due to a feeling of anxiousness, and the likelihood of making the same table choice in the real-world using a 5-point Likert scale. After completing all four treatments, participants are re-administered the Frustration Discomfort Scale to understand any post-study changes in frustration and discomfort, NASA Task Load Index to measure workload, System Usability Scale for perceived usability, and the Virtual Reality Sickness Questionnaire to assess any simulator sickness experienced by the participant. Additionally, we also record head, left-hand, and right-hand position and orientation, and eye-gaze data from the VR headset. Our dataset enables researchers to understand how participant-reported anxiety using the State Trait Anxiety Inventory changes between treatments when compared to the baseline provided before the immersion. Participant-reported loneliness from the Revised UCLA-20 in conjunction with the wait time for the maître d can be used by researchers to understand how decisions to dine in or take out change. Participant eye movements can be studied prior to, during, and after participants opt to dine in or take out to understand whether differences in fixations and saccades are present for participants who opt to dine in versus those that choose take out. The dataset can be used in conjunction with pre-trained times series-based deep learning models such as TEMPO, a Transformer-based model, to predict participant responses after each treatment, such as the closeness of people, noise levels, frustration due to the delay, isolation, and anxiousness.

Specifications Table

**Table:** 

Subject	Computer Sciences
Specific subject area	Understanding participant choices to dine in or take out in an interactive immersive virtual reality café.
Type of data	Table (Comma-Separated Value or CSV) Raw (Comma-Separated Value or CSV)
Data collection	The environment was created using Unity 6000.0.53f1. Characters were created Reallusion Character Creator 4 and animated using Reallusion iClone 8. Sounds were generated using ElevenLabs. All participants provided data using a Meta Quest Pro. All participants completed four treatments, namely no wait for maître d' and no wait for food, no wait for maître d' and 3-minute wait for food, 3-minute wait for maître d' and no wait for food, and 3-minute wait for maître d' and 3-minute wait for food. Participants were randomly assigned to each treatment. All participants were required to be 18 years or older. Participants were excluded if they had a visual acuity deficit >20/200 with corrective lenses, or had a history of motion sickness, had finger injuries, or belonged to vulnerable populations. Participants completed standardized questionnaires, namely Frustration Distraction Scale, Revised UCLA-20 Loneliness Scale, Social Phobia Inventory, State Trait Anxiety Inventory, NASA Task Load Index, System Usability Scale, and Virtual Reality Sickness Questionnaire. The dataset is released under an Apache-2.0 license.
Data source location	Collection Location: Wright State University, Dayton, Ohio, USA Storage: GitHub repository
Data accessibility	Repository name: GitHub Data identification number: https://doi.org/10.5281/zenodo.19175226 Direct URL to data: https://github.com/Terascale-All-sensing-Research-Studio/Dine-In-or-Take-Out-Cafe-Dataset
Related research article	None

## 1 Value of the Data

• The dataset enables researchers in psychology and human behaviour studies to evaluate how self-reported participant loneliness scores relate to choices made by participants in VR environments that simulate typically social environments such as restaurants. The multi-faceted data, which includes interactions with various levels of delay in service, enables researchers to conduct more detailed analysis of loneliness and particular preferences, enabling advancing knowledge over prior observation-based studies on how loneliness influences tasks-oriented decisions, especially among younger individuals.
• By providing data from multiple scales on social anxiety, i.e., the State-Trait Anxiety Inventory (STAI) and the Social Phobia Inventory (SPIN), the dataset enables researchers in psychology to conduct investigation of how reports of general social anxiety relate to choices made in service provision.
• The multi-modal spatiotemporal objective data in the form of eye gaze, head movement, and hand motion data enables researchers to perform correlations between participant reports on loneliness and anxiety, and interactions performed in the VR environment. The spatiotemporal tracks also enable detailed investigation of preferences by facilitating use of techniques such as cluster analysis to identify time spent at key targets in the VR environments, such as non-playable characters and objects of interest, and relate these details to standardized scale and per-treatment responses. Knowledge from these analyses can help inform practitioners on how individuals with loneliness and/or social anxiety can manage interactions in typically social environments.
• The dataset also allows researchers to conduct correlations across subject-reported data on anxiety and objective data such as gaze location and body movement to understand how participants with various anxiety measures respond to content presented in VR.
• Researchers in computer science can use the fine-grained tracked data of gaze, head, and hand motion to develop AI algorithms that automatically recognize participant responses in VR service provision. The spatiotemporal data provided in the dataset enables use of time series-based deep learning models anchored on Transformer. In particular, the data is directly usable for fine-tuning pre-trained time series models such as TEMPO to perform tasks such as recognition of per-trial responses on frustration, isolation, and anxiousness.
• Researchers in human-computer interaction (HCI) and in technology & human behaviour can develop applications that use gaze tracks, head movements, and hand motions to understand participant preferences and perform adaptive generation of content such as VR games or VR productivity apps personalized to user preferences for enhanced engagement in service-oriented VR environments.

## 2 Background

Young adults who fall into the Generation Z, or Gen Z, category are considered the loneliest generation [1]. Prior studies show that loneliness is associated with unhealthy eating habits and sedentary behavior among college-aged US adults [2]. Loneliness among young adults has been found to be associated with social anxiety [3] and enhanced risk of social isolation [4]. As a result, for Gen Z, traditional activities such as eating out as a group in restaurants and cafés have transformed into solo eating activities or opting for take-out services [5]. In this work, we create an immersive virtual reality (VR) application to collect, to the best of our knowledge, a first-of-its-kind dataset on the behaviors of young college-aged adults in a digital twin of a café. We use a café as it provides an environment where individuals go to satisfy basic needs, such as getting food, or to engage in social interactions. Our dataset enables the study of how young adults with varying levels of frustration, loneliness, social phobia, and anxiety engage in a typical scenario of ordering and waiting for food at a café and the choices they make to either order food for dine in or take out. While VR has been used to explore loneliness among young adults, it has been targeted towards gameplay or social platforms [6,7] as opposed to behavior in digital twins of real-world social scenarios.

## 3 Data Description

### 3.1 DATASET overview

Our Dine In or Take Out Dataset can be accessed by visiting our GitHub repository [8]. The dataset consists of 35 participants, of whom 33 fall into the Gen Z, 1 in the cusper Zillennials generation, and 1 from Gen X, interacting in a digital twin of a café to order food for dine in or take out. Our virtual café, called Flavor and Vine, was designed using Unity 6000.0.53f1. Each participant interacts with a maître d' non-playable character (NPC) to order food off a digital menu. Each participant completes the following 4 treatments, with treatments assigned in random order: No wait for maître d' with No wait for food (coded as 00), No wait for maître d' with 3-minute wait for food (coded as 03), 3-minutes wait for maître d' with No wait for food (coded as 30), and 3-minutes wait for maître d' with 3-minute wait for food (coded as 33). In our dataset all participants completed all four treatments and provided data using the same Meta Quest Pro headset. Prior to interacting in the virtual café, we collected participant demographics for age, self-identified gender, ethnicity, race, education level, whether they wear glasses, if they wear contacts, how frequently they play video games, what type of video games they play, how frequently they use VR, if they own a VR device, what type of VR devices they use, how frequently they use take-out services, how frequently they use dine-in services, and which of these services they prefer. All participants completed the Frustration Distraction Scale (FDS) [9], Revised UCLA-20 Loneliness Scale (R-UCLA) [10], Social Phobia Inventory (SPIN) [11], State Trait Anxiety Inventory (STAI) [12] to measure frustration and discomfort intolerance, loneliness, social phobia, and anxiety respectively prior to immersion. After each treatment, participants completed the STAI to record changes in anxiety and completed a survey asking them how close they felt the other people were in the café, how they perceived the noise level, how likely they were to make the same table selection in real-life, and their frustration with the delay, sense of isolation, and feeling of anxiousness. After completing all four treatments, participants completed a second FDS to record changes in frustration as well as the NASA Task Load Index (NASA TLX) [13] to measure perceived workload, System Usability Scale (SUS) [14] to measure overall usability, and the Virtual Reality Sickness Questionnaire (VRSQ) [15] to measure any feelings of simulator discomfort. Each participant provided data in a single session lasting approximately 1 h. For each participant, we used the Meta XR Core SDK in Unity for Meta Quest Pro to acquire head, eye-gaze, left hand, right hand, and button-log data. For each participant we recorded: a button log showing their dialogue and menu choices, eye gaze hit locations showing objects viewed per timestamp based on the gaze ray intersections with scene objects, hand grab log showing the objects interacted with using their hands, headset position and orientation, and left and right-hand position and orientation. In Table 1, we provide the structure of how our dataset is organized in our repository. All folders in our repository contain README.md files that describe the contents of each folder and file within the directory. In our dataset the Participant ID is consistent across all folders.

**Table 1:** GitHub folder structure. CSV = Comma separated values. The contents of the table below were generated by going through each folder and subfolder in the GitHub repository of the dataset. Link: https://github.com/Terascale-All-sensing-Research-Studio/Dine-In-or-Take-Out-Cafe-Dataset/tree/main.

FolderName	SubFolders	DataType	Contents
Assets	Yes	MP3	Contains two subfolders, namely 00_03_Audio_Files and 30_33_Audio_Files. The folder 00_03_Audio_Files contains all the audio assets in MP3 format for the No wait for maître d' with No wait for food and No wait for maître d' with 3-minute wait for food treatments. 30_33_Audio_Files contains all the audio assets in MP3 format for the 3-minute wait for maître d' with No wait for food and 3-minute wait for maître d' with 3-minute wait for food treatments. The audio files are generated using ElevenLabs.
Cybersickness	None	CSV	Participant responses to the standard Virtual Reality Cyber Sickness Questionnaire (VRSQ). The CSV files are prefixed as numerical_ and string_ to indicate the format of the scale values. The unique 5-character ID is the same as the one found in the Demographics folder.
Demographics	None	CSV	Participant demographics consisting of: age, self-identified gender, ethnicity, race, education level, whether they wear glasses, if they wear contacts, how frequently they play video games, what type of video games they play, how frequently they use VR, if they own a VR device, what type of VR devices they use, how frequently they use take-out services, how frequently they use dine-in services, and which of these services they prefer. Each participant is assigned a unique 5-character ID.
FDS	None	CSV	Participant responses to the standard Frustration Discomfort Scale (FDS). The CSV files are annotated as pre_ and post_ to indicate that they were administered prior to and after the immersion. The CSV files are prefixed as numerical_ and string_ to indicate the format of the scale values. The unique 5-character ID is the same as the one found in the Demographics folder.
NASA_TLX	None	CSV	Participant responses to the standard 21-tick NASA Task Load Index (TLX). The unique 5-character ID is the same as the one found in the Demographics folder.
ObjectNames	None	None	The EyeGaze.csv found in the VRData folder for each participant contains a column titled objectName that stores the name of the object being observed. A top-down view of the scene is provided along with a list of labeled ObjectNames.
R_UCLA	None	CSV	Participant responses to the Revised UCLA Loneliness Scale (R-UCLA). The CSV files are prefixed as numerical_ and string_ to indicate the format of the scale values. The unique 5-character ID is the same as the one found in the Demographics folder.
SPIN	None	CSV	Participant responses to the Social Phobia Inventory (SPIN). The CSV files are prefixed as numerical_ and string_ to indicate the format of the scale values. The unique 5-character ID is the same as the one found in the Demographics folder.
STAI	None	CSV	Participant responses to the State Trait Anxiety Inventory (STAI). The CSV files are prefixed as numerical_ and string_ to indicate the format of the scale values. The unique 5-character ID is the same as the one found in the Demographics folder.
SUS	None	CSV	Participant responses to the standard System Usability Scale (SUS). The CSV files are prefixed as numerical_ and string_ to indicate the format of the scale values. The unique 5-character ID is the same as the one found in the Demographics folder.
TreatmentResponses	None	CSV	Participant responses to each treatment, i.e., not being forced to wait for a social interaction (00 or 03) or being forced to wait for a social interaction (33 or 30). Participant responses include their frustration level on a 5-point Likert scale caused by people proximity, noise level, time delay, feeling isolated, anxiousness, and finally their likelihood of making the same choices in real life. The CSV files are prefixed as numerical_ and string_ to indicate the format of the scale values. The unique 5-character ID is the same as the one found in the Demographics folder.
VRData	Yes	CSV	Contains 35 subfolders that represents each participant. Each subfolder is named with the unique 5-character ID. The unique 5-character ID is the same as the one found in the Demographics folder. Within each participant subfolder there are four additional subfolders named ParticipantID_0_0, ParticipantID_0_3, ParticipantID_3_0, and ParticipantID_3_3. Here Participant_ID is the unique 5-character ID and _0_0, _0_3, _3_0, and _3_3 are the wait conditions. Within the ParticipantID_0_0, ParticipantID_0_3, ParticipantID_3_0, and ParticipantID_3_3 subfolders are *six CSV files for Button Log (ButtonLog), Eye Gaze (EyeGazeLog), *Hand Grab Log (HandGrabLog), Head Position and Orientation (Head), Left Hand (LeftHand), and Right Hand (RightHand). Thus, each participant has *24 CSV files. * HandGrabLog is only created if a participant interacts with an object in the environment using their hands.

### 3.2 Directory tree

We provide the directory tree below for the repository and the tree for one example participant (0P9C0) in the VRData folder:

**Table:** 

### 3.3 DATA dictionary

The dictionary.csv file summarizes the data found in the following CSV files in our dataset:

• Cybersickness/numerical_vrsq.csv
• Cybersickness/string_vrsq.csv
• Demographics/demographics.csv
• FDS/numerical_pre_fds.csv
• FDS/numerical_post_fds.csv
• FDS/string_pre_fds.csv
• FDS/string_post_fds.csv
• NASA_TLX/nasatlx.csv
• R_UCLA/numerical_ucla.csv
• R_UCLA/string_ucla.csv
• SPIN/numerical_spin.csv
• SPIN/string_spin.csv
• STAI/numerical_00_stai.csv
• STAI/numerical_03_stai.csv
• STAI/numerical_30_stai.csv
• STAI/numerical_03_stai.csv
• STAI/numerical_33_stai.csv
• STAI/numerical_pre_stai.csv
• STAI/string_00_stai.csv
• STAI/string_03_stai.csv
• STAI/string_30_stai.csv
• STAI/string_33_stai.csv
• STAI/string_pre_stai.csv
• SUS/numerical_sus.csv
• SUS/string_sus.csv
• Treatment Responses/numerical_00_responses.csv
• Treatment Responses/numerical_03_responses.csv
• Treatment Responses/numerical_30_responses.csv
• Treatment Responses/numerical_33_responses.csv
• Treatment Responses/string_00_responses.csv
• Treatment Responses/string_03_responses.csv
• Treatment Responses/string_30_responses.csv
• Treatment Responses/string_33_responses.csv
• Treatment Responses/treatment_order.csv

We provide a summary of the dictionary.csv file contents in Table 2. The dictionary.CSV file can be used to obtain headers for the non VRData CSV files in our dataset.

**Table 2:** The data dictionary file dictionary.csv summarizes the contents of the CSV files found within Cybersickness, Demographics, FDS, NASA_TLX, R_UCLA, SPIN, STAI, SUS, and TreatmentResponses folders. Link: https://github.com/Terascale-All-sensing-Research-Studio/Dine-In-or-Take-Out-Cafe-Dataset/blob/main/dictionary.csv.

Column	Description
Directory/FileName	Provides the directory and filename, e.g., Demographics/demographics.csv
ColumnName	Provides the column name in the file being referenced in Directory/FileName, e.g., Participant ID
Value	Provides detailed information of the value stored in ColumnName, e.g., Unique 5-character ID assigned to each participant

### 3.4 Dataset frame rate summary

We summarize the minimum (Min), maximum (Max), and average (Mean) frame rate in Table 3 for the eye gaze, head, left hand, and right hand for our dataset. The summary is provided as frames per second by iterating over the VRData folder using the script vrdata_frame_summary.py.

**Table 3:** We provide the minimum (Min), maximum (Max), average (Mean), and standard deviation (SD) for the eye gaze, left hand, and right-hand data by using the Python script vrdata_frame_summary.py and interating over the data stored in the VRData folder of the GitHub repository. Link: https://github.com/Terascale-All-sensing-Research-Studio/Dine-In-or-Take-Out-Cafe-Dataset/blob/main/vrdata_frame_summary.py.

Treatment	Type of Data	Min	Max	Mean	SD
00	EyeGaze	46.69	70.72	66.39	4.89
	Head	46.83	70.45	66.85	4.70
	LeftHand	46.83	70.45	66.85	4.70
	RightHand	46.83	70.45	66.85	4.70
03	EyeGaze	50.75	71.67	68.78	3.80
	Head	52.42	71.71	70.00	3.29
	LeftHand	52.42	71.71	70.00	3.29
	RightHand	52.42	71.71	70.00	3.29
30	EyeGaze	42.72	71.40	68.13	5.75
	Head	43.79	71.43	69.41	5.32
	LeftHand	43.79	71.43	69.41	5.32
	RightHand	43.79	71.43	69.41	5.32
33	EyeGaze	51.64	71.46	69.09	3.51
	Head	52.60	72.45	70.31	3.32
	LeftHand	52.60	72.45	70.31	3.32
	RightHand	52.60	72.45	70.31	3.32

### 3.5 Folder: assets

The audio assets for the 00 and 03 treatments are stored in the 00_03_Audio_Files subfolder, while the ones for the 30 and 33 treatments are stored in the 30_33_Audio_Files subfolder. The audio files are generated using ElevenLabs and stored in MP3 format. In Table 4, we describe how each audio asset is used in each treatment. We use **Present** to indicate the asset is used and **Absent** to indicate the asset is not used for the particular treatment.

**Table 4:** Audio assets used in each treatment are generated using ElevenLabs. Present indicates that the audio asset was used and Absent indicates that it was not used in the treatment. 00, 03, 30, and 33 are the abbreviations for the treatments. Link: https://github.com/Terascale-All-sensing-Research-Studio/Dine-In-or-Take-Out-Cafe-Dataset/tree/main/Assets.

Audio Asset Name	00	03	30	33
WithYouShortly.mp3	Absent	Absent	Present	Present
Welcome.mp3	Present	Present	Present	Present
WelcomeLoop.mp3	Present	Present	Present	Present
OkRightThisWay.mp3	Present	Present	Present	Present
WillStandingTableWork.mp3	Present	Present	Present	Present
WillTakeoutWork.mp3	Present	Present	Present	Present
Sorry.mp3	Present	Present	Present	Present
FemaleBackgroundConvo.mp3	Present	Present	Present	Present
TrimmedCafeAmbientNoise.mp3	Present	Present	Present	Present

### 3.6 Folder: cybersickness

We administered the standard Virtual Reality Sickness Questionnaire (VRSQ) to each participant after they completed all four treatments. We used the VRSQ to measure whether participants experienced any simulator sickness after completing the four treatments. Participants provide their responses using a 4-point Likert Scale, where 1 is None, 2 is Slight, 3 is Moderate, and 4 is Severe. An example question from the VRSQ is: “General Discomfort”. The numerical_ and string_ prefixes are used to indicate the format of the scale values. The CSV file contains raw data and has not been processed.

### 3.7 Folder: demographics

We store participant demographics in the file demographics.csv. The CSV file contains raw data and has not been processed.

A summary of our participant demographics are provided as follows:

• Age: Minimum = 19, Maximum = 53, Average = 24.8.
• Race: American Indian or Alaska Native = 1, Asian = 18; White = 15, Two or more Races = 1.
• Self-Identified Gender: Male = 26, Female = 9.
• Education Level: High School or Equivalent = 9, Associates Degree = 3, Bachelor's Degree = 17, Master's Degree = 5, Other = 1.
• Glasses: No = 19, Yes = 16.
• VR usage: Never = 21, Rarely = 9, Sometimes = 4, Often = 1.
• Take-out service frequency: Never = 1, Rarely = 6, Sometimes = 18, Often = 9, Always = 1.
• Dine-in service frequency: Rarely = 5, Sometimes = 17, Often = 13.
• Dining preference: Dine in = 12, Take out = 13, No preference = 10.

### 3.8 Folder: FDS

We administered the standard Frustration Discomfort Scale (FDS) at the start of the study and after participants had completed all four treatments. We used FDS to understand frustration intolerance before and after immersion into the café. Participants provide their response using a 5-point Likert Scale, where 1 is Absent, 2 is Mild, 3 is Moderate, 4 is Strong, and 5 is Very Strong. An example question from the FDS is: “I can’t stand doing things that involve a lot of hassle”. The files numerical_pre_fds.csv and string_pre_fds.csv contain the responses from each participant prior to the treatments, while the files numerical_post_fds.csv and string_post_fds.csv contains responses after completion of all four treatments. The numerical_ and string_ prefixes are used to indicate the format of the scale values. The CSV file contains raw data and has not been processed.

### 3.9 Folder: NASA_TLX

We use the standard 21-tick version of the NASA TLX at the end of the study and automatically scaled the scores from 0 to 100. We used NASA TLX to understand participant workload when interacting within the immersive café. An example question from NASA TLX is: “Mental Demand: How mentally demanding was the task?” The file nasatlx.csv contains responses from each participant. The CSV file has raw data and is not processed.

### 3.10 Folder: objectNames

**Fig. 1:**
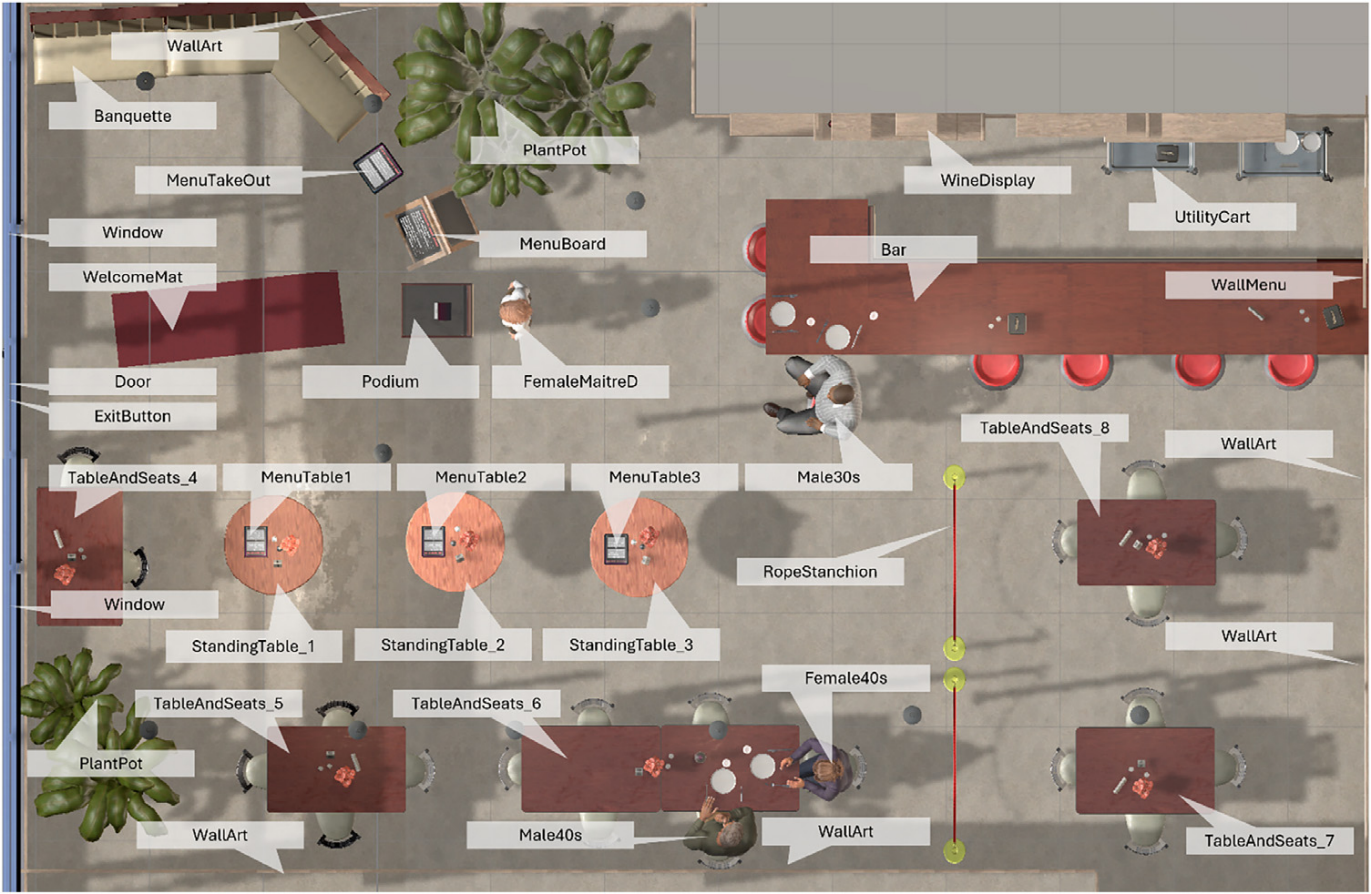
Top down view image of the scene showing labeled ObjectNames. Not all ObjectNames are visible in the image. Link: https://github.com/Terascale-All-sensing-Research-Studio/Dine-In-or-Take-Out-Cafe-Dataset/tree/main/ObjectNames.

In the folder ObjectNames we provide a list of all object names stored in the objectName column of the EyeGaze.csv file in the VRData folder for each participant. As illustrated in Fig. 1, we provide a top-down view of the scene with the ObjectNames for each object.

The following ObjectNames are visible and labeled in the bird’s eye view image:

• Banquette
• Bar
• Door
• ExitButton
• Female40s
• FemaleMaitreD
• Male30s
• Male40s
• MenuBoard
• MenuTable1
• MenuTable2
• MenuTable3
• MenuTakeOut
• NapkinDispenser
• PlantPot
• Podium
• RopeStanchion
• StandingTable_1
• StandingTable_2
• StandingTable_3
• TableAndSeats_4
• TableAndSeats_5
• TableAndSeats_6
• TableAndSeats_7
• TableAndSeats_8
• UtilityCart
• WallArt
• WallMenu
• WelcomeMat
• WineDisplay
• Window

The following ObjectNames are not labeled in the image as they are too small to see, however, the names are descriptive to the object:

• Barstool_0
• Barstool_1
• Barstool_2
• Barstool_3
• Barstool_4
• Barstool_5
• Barstool_6
• Fork
• Fork_Bar
• Knife
• Knife_Bar
• Lamp
• NapkinDispenser
• NapkinDispenser_UtilityCart
• NumberSign_1
• NumberSign_2
• NumberSign_3
• NumberSign_4
• NumberSign_5
• NumberSign_6
• NumberSign_7
• NumberSign_8
• PepperShaker_4
• PepperShaker_5
• PepperShaker_6
• PepperShaker_7
• PepperShaker_8
• PepperShaker_Bar
• PepperShaker_StandingTable_1
• Plate_6
• Plate_Bar
• ReservedSign_4
• ReservedSign_5
• ReservedSign_7
• ReservedSign_8
• ReservedSign_Bar
• SaltShaker_4
• SaltShaker_5
• SaltShaker_6
• SaltShaker_7
• SaltShaker_8
• SaltShaker_Bar
• SaltShaker_StandingTable_1
• TabletStand_StandingTable1
• TabletStand_StandingTable2
• TabletStand_StandingTable3
• TabletStand_TakeOut
• Tablet_Podium
• Vase_1
• Vase_2
• Vase_3
• Vase_4
• Vase_5
• Vase_6
• Vase_7
• Vase_8
• WineBottle_6
• WineGlass_Bar
• WineGlass_Table_6

The following ObjectNames represent the parts of the ceiling in the scene. They are not labeled in the image; however, the suffix (e.g. _Entrance) provides a mapping to the ObjectName in the bird’s eye view image. Thus, in this example, the ObjectName Ceiling_Entrance represents the ceiling directly above the welcome mat in the scene.

• Ceiling
• CeilingPipe
• CeilingVent
• CeilingVentSupport
• Ceiling_Bar
• Ceiling_Entrance
• Ceiling_Hallway
• Ceiling_Hostess
• Ceiling_Table_6
• Ceiling_WaitingBooth

The following ObjectNames represent the floor. They are not labeled in the image, however, the suffix (e.g. _Table_6) provides a mapping to the ObjectName in the bird’s eye view image. Thus, in this example, the ObjectName Floor_Table_6 represents the floor directly below TableAndSeats_6 in the scene.

• ExteriorGround
• ExteriorHall
• Floor_Bar
• Floor_Entrance
• Floor_Hallway
• Floor_Podium
• Floor_StandingTable_1
• Floor_StandingTable_2
• Floor_StandingTable_3
• Floor_Table_5_6
• Floor_Table_5_Plants
• Floor_Table_6
• Floor_Table_8
• Floor_WaitingBooth
• Floor_WaitingBooth_Plants

### 3.11 Folder: R_UCLA

Prior to immersion, we administered the Revised UCLA Loneliness Scale to each participant. We used the Revised UCLA Loneliness Scale to understand how participant perceived their own subjective feelings of loneliness. Participants provide their response using a 4-point Likert Scale, where 1 is Never, 2 is Rarely, 3 is Sometimes, and 4 is Often. An example question from the Revised UCLA Loneliness Scale is: “I feel in tune with the people around me”. The files numerical_ucla.csv and string_ucla.csv contains participant responses. The numerical_ and string_ prefixes are used to indicate the format of the scale values. The CSV file contains raw data and has not been processed.

### 3.12 Folder: SPIN

Prior to immersion, we administered the Social Phobia Inventory (SPIN) to each participant. We used the Social Phobia Inventory to understand how participant perceived their own subjective feelings with regards to social phobias. Participants provide their response using a 5-point Likert Scale, where 1 is Not at all, 2 is A little, 3 is Somewhat, 4 is Very much, and 5 is Extremely. An example question from the Social Phobia Inventory is: “I am afraid of people in authority”. The files numerical_spin.csv and string_spin.csv contains participant responses. The numerical_ and string_ prefixes are used to indicate the format of the scale values. The CSV file contains raw data and has not been processed.

### 3.13 Folder: STAI

The State Trait Anxiety Inventory (STAI) was administered to every participant throughout the study a total of five times:

1. Pre-STAI: taken before the participant is exposed to treatments.
2. No wait for maître d' with No wait for food (00): The participant has no wait time for the maître d' NPC's interaction and no wait time after the menu selection.
3. No wait for maître d' with 3-minute wait for food (03): The participant has no wait time for the maître d' NPC's interaction, but a 3-minute wait time after the menu selection.
4. 3-minutes wait for maître d' with No wait for food (30): The participant has a 3-minute wait time for the maître d' NPC's interaction and no wait time after the menu selection.
5. 3-minutes wait for maître d' with 3-minute wait for food (33): The participant has a 3-minute wait time for the maître d' NPC's interaction and a 3-minute wait time after the menu selection.

We used the State Trait Anxiety Inventory to understand participant feelings of anxiety. Participants provide their response using a 4-point Likert Scale, where 1 is Not at all, 2 is A little, 3 is Somewhat, and 4 is Very much so. An example question from the State Trait Anxiety Inventory is: “I feel calm”. The files numerical_00_stai.csv, numerical_03_stai.csv, numerical_30_stai.csv, numerical_33_stai.csv, numerical_pre_stai.csv, string_00_stai.csv, string_03_stai.csv, string_30_stai.csv, string_33_stai.csv, and string_pre_stai.csv contain raw data in CSV format from each participant. The numerical_ and string_ prefixes are used to indicate the format of the scale values.

### 3.14 Folder: SUS

The standard System Usability Scale (SUS) was administered at the end of the study. We used the SUS to understand how participants perceived the overall usability of the immersive café. Participants provide their response using a 5-point Likert Scale, where 5 is Strongly agree, 4 is Somewhat agree, 3 is Neither agree nor disagree, 2 is Somewhat disagree, and 1 is Strongly disagree. An example question from the System Usability Scale is: “I would like to use this system frequently.”. The files numerical_sus.csv and string_sus.csv contains raw data in CSV format from each participant. The numerical_ and string_ prefixes are used to indicate the format of the scale values.

### 3.15 Folder: treatmentresponses

Our study consisted of four treatments:

1. **No wait for maître d' with No wait for food (coded as 00):** The participant is spawned into the cafe area and may approach the maître d' podium at any time to engage in dialogue with the maître d' NPC. The participant may then make a selection on a menu item, and the scene ends.
2. **No wait for maître d' with 3-minute wait for food (coded as 03):** The participant is spawned into the cafe area and may approach the maître d' podium at any time to engage in dialogue with the maître d' NPC. The participant may then make a selection on a menu item, starting a 3-minute wait for food. The scene ends after the wait time.
3. **3-minutes wait for maître d' with No wait for food (coded as 30)**: The participant is spawned into the cafe area and may approach the maître d' podium at any time. The interacting maître d' NPC tells the participant she will be with them shortly, starting a 3-minute wait time. After 3-minutes, the maître d' engages in dialogue with the participant. The participant may then make a selection on a menu item, and the scene ends.
4. **3-minutes wait for maître d' with 3-minute wait for food (coded as 33):** The participant is spawned into the cafe area and may approach the maître d' podium at any time. The interacting maître d' NPC tells the participant she will be with them shortly, starting a 3-minute wait time. After 3-minutes, the maître d' engages in dialogue with the participant. The participant may then make a selection on a menu item, starting a 3-minute wait for food. The scene ends after the wait time.

The file treatment_order.csv contains the random order participants were given each of the four treatments. Table 5 provides the data overview of the file format.

**Table 5:** Treatment order by Participant ID is stored in the treatment_order.csv file in the TreatmentResponses folder in the GitHub repository. Link: https://github.com/Terascale-All-sensing-Research-Studio/Dine-In-or-Take-Out-Cafe-Dataset/tree/main/TreatmentResponses.

Dataset Column	Data	Response Value
1	Participant ID	Unique 5-character ID assigned to each participant.
2	Treatment1	00, 03, 30, or 33 to indicate which treatment the participant completed first.
3	Treatment2	00, 03, 30, or 33 to indicate which treatment the participant completed second.
4	Treatment3	00, 03, 30, or 33 to indicate which treatment the participant completed third.
5	Treatment4	00, 03, 30, or 33 to indicate which treatment the participant completed fourth.

The files string_03_responses.csv, string_30_responses.csv, and string_33_responses.csv contains participant responses in string format, whereas the files numerical_00_responses.csv, numerical_03_responses.csv, numerical_30_responses.csv, numerical_33_responses.csv, string_00_responses.csv contain the responses in numerical format. The CSV files contain participant responses to the following questions after each treatment:

1. “I felt the people in the cafe were…”. We use a 5-point Likert Scale with options from Very close (1), Somewhat close (2), Neither (3), Somewhat far (4), and Very far (5).
2. “I felt the noise level in the cafe was… ” We use a 5-point Likert Scale with options from Very quiet (1), Somewhat quiet (2), Neither (3), Somewhat loud (4), and Very loud (5).
3. “What was your frustration level due to length of delay?” We use a 5-point Likert scale with options from Very low (1), Somewhat low (2), Neither (3), Somewhat high (4), and Very high (5).
4. “What was your frustration level due to feelings of isolation?” We use a 5-point Likert scale with options from Very low (1), Somewhat low (2), Neither (3), Somewhat high (4), and Very high (5).
5. “What was your frustration level due to feeling anxious?” We use a 5-point Likert scale with options from Very low (1), Somewhat low (2), Neither (3), Somewhat high (4), and Very high (5).
6. “How likely are you to make the same table selection in real life? “ We use a 5-point Likert Scale with options from Very likely (1), Somewhat likely (2), Neither (3), Somewhat unlikely (4), and Very unlikely (5).

The file contains the data described in Table 6.

**Table 6:** Post treatment, i.e. 00, 03, 30, and 33, responses are stored in numerical and string formats of the files 00_responses.csv, 03_responses.csv, 30_responses.csv, and 33_responses.csv in the TreatmentResponses folder in the GitHub repository. The responses include the participant perception of people proximity, noise level, delay, isolation, anxiety, and real-life likelihood. Link: https://github.com/Terascale-All-sensing-Research-Studio/Dine-In-or-Take-Out-Cafe-Dataset/tree/main/TreatmentResponses.

Dataset Column	Data	Response Value
1	Participant ID	Unique 5-character ID assigned to each participant
2	TreatmentPeopleProximity	String or Integer Likert scale value with 1 being Very close, 2 being Somewhat close, 3 being Neither, 4 being Somewhat far, and 5 being Very far, indicating the participant's perception of proximity of the NPC's in the scene
3	TreatmentNoiseLevel	String or Integer Likert scale value with 1 being Very quiet, 2 being Somewhat quiet, 3 being Neither, 4 being Somewhat loud, and 5 being Very loud, indicating the participant's perception of noise level of the scene.
4	TreatmentDelay	String or Integer Likert scale value with 1 being Very low, 2 being Somewhat low, 3 being Neither, 4 being Somewhat high, and 5 being Very high, indicating the participant's frustration level with delay times.
5	TreatmentIsolation	String or Integer Likert scale value with 1 being Very low, 2 being Somewhat low, 3 being Neither, 4 being Somewhat high, and 5 being Very high, indicating the participant's frustration level from feeling isolated.
6	TreatmentAnxiety	String or Integer Likert scale value with 1 being Very low, 2 being Somewhat low, 3 being Neither, 4 being Somewhat high, and 5 being Very high, indicating the participant's frustration level from feeling anxious.
7	TreatmentRealLifeLikelihood	String or Integer Likert scale value with 1 being Very likely, 2 being Somewhat likely, 3 being Neither, 4 being Somewhat unlikely, and 5 being Very unlikely, indicating the participant's likeliness to make the same choices in a similar real-life scenario.

### 3.16 FOLDER: VRData

A total of 35 subfolders, each representing a single participant, can be found in the VRData folder in our repository. The 35 subfolders are named with the unique 5-character ID assigned to each participant during the collection. Within each participant subfolder are four sub-subfolders representing the four treatments. These sub-subfolders are named ParticipantID_0_0, ParticipantID_0_3, ParticipantID_3_0, and ParticipantID_3_3, where ParticipantID is the 5-character ID assigned to each participant. The suffix _0_0, _0_3, _3_0, and _3_3 indicates the four treatments, namely whether the participant was in a no wait for maître d' with no wait for food (_0_0), a no wait for maître d' with 3-minute wait for food (_0_3), a 3-minute wait for maître d' with no wait for food (_3_0), or a 3-minute wait for maître d' with 3-minute wait for food treatment (_3_3). Within the 0_0, 0_3, 3_0, and 3_3 sub-subfolders are at minimum 5 raw CSV files, for a total of 20 raw CSV files per participant. An additional 6th raw CSV file is included only if the participant interacts with objects in the scene using their hands, with a max possible total of 24 raw CSV files per participant. The names and locations of each of these 5 CSV files are described in Table 7.

**Table 7:** Data folder locations of data captured using the Meta Quest Pro. Each CSV file is located within a participant-based subfolder in the VRData subfolder in the GitHub repository. A * prefix before a file indicates that the file only exists if the participant interacted with objects in the scene using their hands. Link: https://github.com/Terascale-All-sensing-Research-Studio/Dine-In-or-Take-Out-Cafe-Dataset/tree/main/VRData.

Filename	Location
ButtonLog.csv	VRData/ParticipantID/ParticipantID_0_0
EyeGaze.csv	
*HandGrabLog.csv	
Head.csv	
LeftHand.csv	
RightHand.csv	
ButtonLog.csv	VRData/ParticipantID/ParticipantID_0_3
EyeGaze.csv	
*HandGrabLog.csv	
Head.csv	
LeftHand.csv	
RightHand.csv	
ButtonLog.csv	VRData/ParticipantID/ParticipantID_3_0
EyeGaze.csv	
*HandGrabLog.csv	
Head.csv	
LeftHand.csv	
RightHand.csv	
ButtonLog.csv	VRData/ParticipantID/ParticipantID_3_3
EyeGaze.csv	
*HandGrabLog.csv	
Head.csv	
LeftHand.csv	
RightHand.csv	

In Table 8, we describe the content stored in each of these 6 CSV files per participant.

**Table 8:** Details of the content stored in each CSV folder for the button log, eye gaze, hand grab log, head, left hand, and right hand. The data is stored for each participant within the VRData folder in the GitHub repository. A * prefix before a file indicates that the file only exists if the participant interacted with objects in the scene using their hands. Link: https://github.com/Terascale-All-sensing-Research-Studio/Dine-In-or-Take-Out-Cafe-Dataset/tree/main/VRData.

Filename	VariableName: Content
ButtonLog.csv	**timeStampNs:** Time stamp in nanoseconds for when the button interaction event was recorded. **gameTime:** Game time stamp in seconds for when the button interaction event was recorded. **buttonSelection:** Human readable name for the button interacted with in the scene.
EyeGaze.csv	**timeStampNs:** Time stamp in nanoseconds for when the eye gaze event was recorded. **gameTime:** Game time stamp in seconds for when the eye gaze event was recorded. **objectName:** Human readable name for the scene object the eye gaze has recorded. **posX:** X position of the eye gaze hit location. **posY:** Y position of the eye gaze hit location. **posZ:** Z position of the eye gaze hit location.
*HandGrabLog.csv	**timeStampNs:** Time stamp in nanoseconds for when the head movement was recorded. **gameTime:** Game time stamp in seconds for when the head movement was recorded. **hand:** Specifies which hand the object was interacted with. **objectName:** Human readable name for the object interacted with in the scene.
Head.csv	**timeStampNs:** Time stamp in nanoseconds for when the head movement was recorded. **gameTime:** Game time stamp in seconds for when the head movement was recorded. **posX:** X position of the head location. **posY:** Y position of the head location. **posZ:** Z position of the head location. **rotX:** X rotation of the head location. **rotY:** Y rotation of the head location. **rotZ:** Z rotation of the head location.
LeftHand.csv	**timeStampNs:** Time stamp in nanoseconds for when the left-hand movement was recorded. **gameTime:** Game time stamp in seconds for when the left-hand movement was recorded. **posX:** X position of the left hand location. **posY:** Y position of the left hand location. **posZ:** Z position of the left hand location. **rotX:** X rotation of the left hand location. **rotY:** Y rotation of the left hand location. **rotZ:** Z rotation of the left hand location.
RightHand.csv	**timeStampNs:** Time stamp in nanoseconds for when the right-hand movement was recorded. **gameTime:** Game time stamp in seconds for when the right-hand movement was recorded. **posX:** X position of the right hand location. **posY:** Y position of the right hand location. **posZ:** Z position of the right hand location. **rotX:** X rotation of the right hand location. **rotY:** Y rotation of the right hand location. **rotZ:** Z rotation of the right hand location.

## 4 Experimental Design, Materials and Methods

**Fig. 2:**
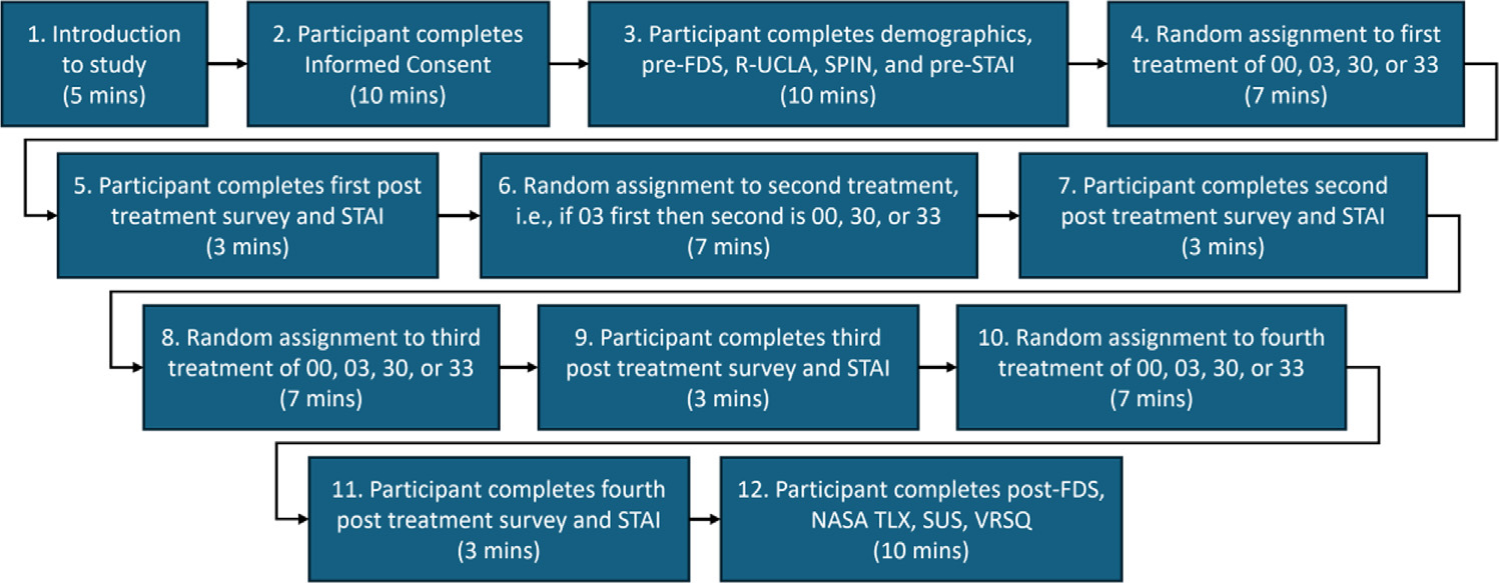
Overall study flow with approximate timing information.

Our data collection was conducted at the Terascale All-sensing Research Studio (TARS) virtual reality (VR) lab at the Dayton, OH, USA campus of Wright State University. The research studio is managed and operated by Dr. Natasha Banerjee, Dr. Sean Banerjee, and Dr. Ashutosh Shivakumar. The data collection was conducted during the Fall 2025 academic semester at the university between the months of November and December. Data collection was conducted after the research team received approval from the Institutional Review Board (IRB) at Wright State University. The dataset was collected using a Meta Quest Pro. The VR application used in the dataset collection was developed using Unity. The final collected dataset consists of 35 participants providing data. Data collection for each participant was conducted in a single session. The session time for each participant ranged from 60-minutes to 75-minutes. During the collection session, each participant interacted in a VR café for four treatments, where each treatment consisted of the NPC maître d' either providing service without delay or informing them of delay of service, in combination with a delay post menu item selection or no delay post menu selection. In Fig. 2, we provide a summary of the data collection process.

### 4.1 Approval

The dataset described in this paper was collected after receiving approval from the Wright State University Institutional Review Board (IRB) under IRB-2025–901. Prior to data collection, all researchers completed the Collaborative Institutional Training Initiative (CITI Program) Human Subjects Research training. The methods described in this paper were performed under the guidelines approved by the IRB.

### 4.2 VR Cafe

**Fig. 3:**
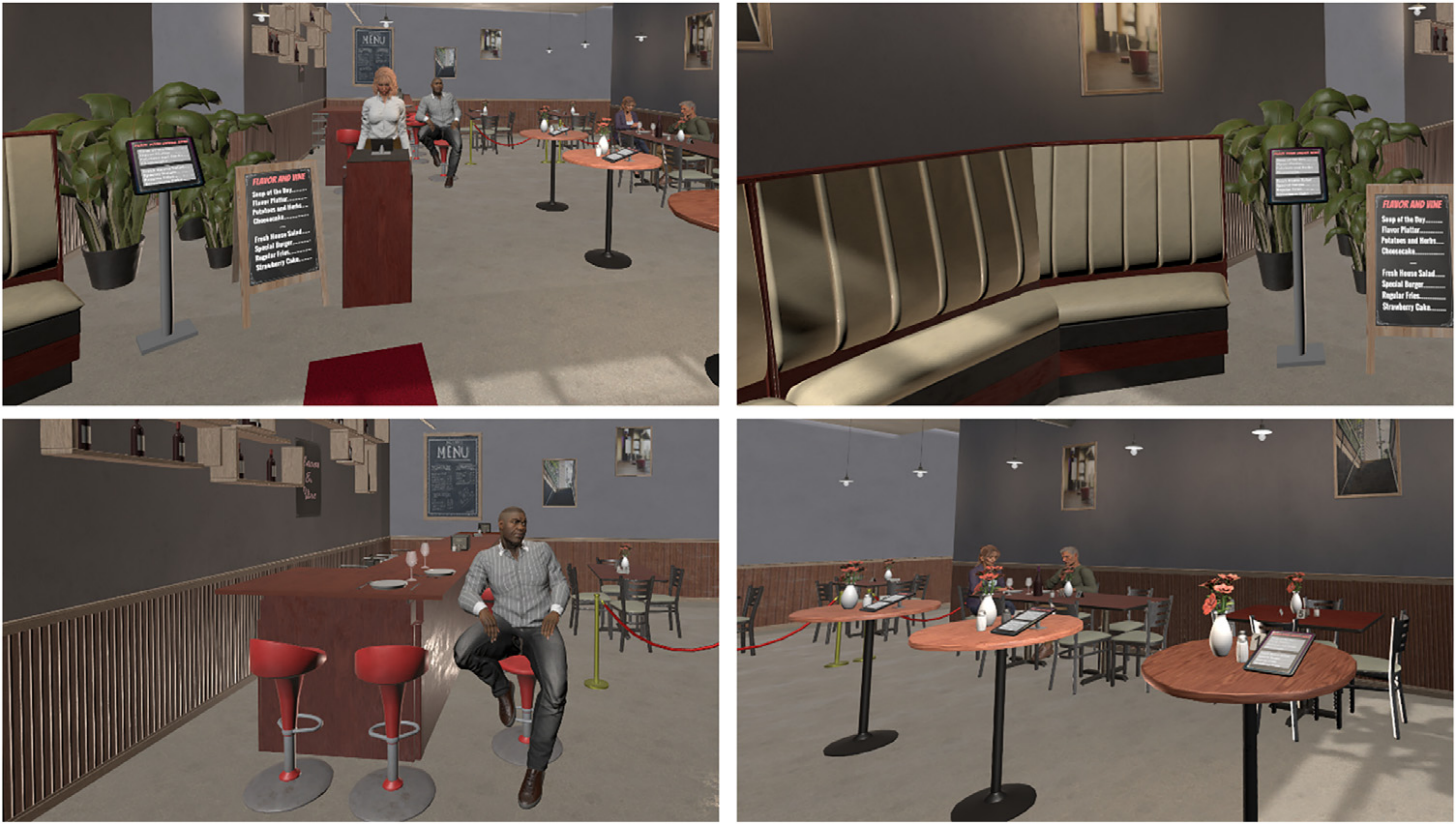
Top left image shows the entryway the participant spawns to. Top right image shows the waiting area. Bottom left image shows the bar counter and rope stanchion as an expansion of the dining area. Bottom right image shows the dining area with seated tables and standing tables.

Our virtual café, Flavor and Vine, was created using Unity 6000.0.53.f1. Data collection was conducted using a single Meta Quest Pro for all participants to minimize any device level variations across participants. Synchronization of the headset, hands, and eye tracking was performed at the device level with no additional synchronization systems being used. When designing the VR application, the native Unity left-handed coordinate system was used with X pointing to the right, Y pointing up, and Z pointing forward. The data is stored on the Meta Quest Pro and directly exported with no transformations, cleanups, or post-processing. We design Flavor and Vine to take the visual appearance of a typical modern urban café. The streetscape outside the café also represents a typical thoroughfare, however, we do not animate the street with moving cars or people. The interior of Flavor and Vine consists of an entryway, a waiting area, and a dining area. The entryway consists of a red carpet that guides the participant to the maître d’ podium where a wooden menu display board informs them of the menu options. As shown in Fig. 3, once the participant is past the entryway, the waiting area consists of a banquette and a digital tablet that serves as the take-out menu. The dining area of Flavor and Vine consists of 5 sitting tables with chairs, 3 standing tables, and a bar area with chairs. Standing Table 1 is placed closest to the entryway, with the other two tables continuing in numerical order further into the dining area. Past standing Table 3 we create a cordoned off area with a rope stanchion with additional seating. We include objects typically found in real world restaurants and cafés such as flower vases, condiments, table numbers on all tables of the dining area. In addition, our seated tables may include reserved signs, plates, cutlery, and wineglasses. On the three standing tables we include a digital tablet that will be used as menus. For further realism, we place potted plants, wall art, wine shelves and bottles on the walls, and metallic vents and lighting fixtures on the ceiling. Once the participant is immersed, we spawn them into the entryway facing the maître d' podium.

### 4.3 Study treatments

Our study consisted of four treatments:

1. **No wait for maître d' with No wait for food (coded as 00):** The participant is spawned into the cafe area and may approach the maître d' podium at any time to engage in dialogue with the maître d' NPC. The participant may then make a selection on a menu item, and the scene ends.
2. **No wait for maître d' with 3-minute wait for food (coded as 03):** The participant is spawned into the cafe area and may approach the maître d' podium at any time to engage in dialogue with the maître d' NPC. The participant may then make a selection on a menu item, starting a 3-minute wait for food. The scene ends after the wait time.
3. **3-minutes wait for maître d' with No wait for food (coded as 30)**: The participant is spawned into the cafe area and may approach the maître d' podium at any time. The interacting maître d' NPC tells the participant she will be with them shortly, starting a 3-minute wait time. After 3-minutes, the maître d' engages in dialogue with the participant. The participant may then make a selection on a menu item, and the scene ends.
4. **3-minutes wait for maître d' with 3-minute wait for food (coded as 33):** The participant is spawned into the cafe area and may approach the maître d' podium at any time. The interacting maître d' NPC tells the participant she will be with them shortly, starting a 3-minute wait time. After 3-minutes, the maître d' engages in dialogue with the participant. The participant may then make a selection on a menu item, starting a 3-minute wait for food. The scene ends after the wait time.

### 4.4 Non-playable characters (NPCs)

**Fig. 4:**
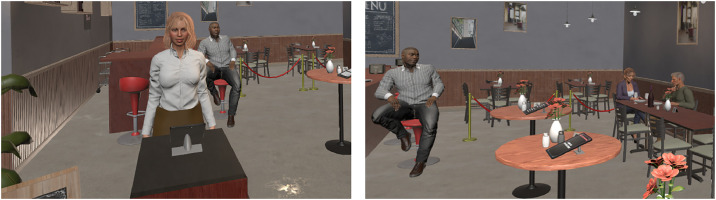
On the left, we show the only interactive maître d' NPC who engages with the participant. On the right, we show the three non-interactive customer NPCs who do not engage with the participant. One male NPC is seated alone at the bar counter, and the other female and male NPCs are seated together at a seated table.

As shown in Fig. 4, our virtual café, Flavor and Vine, consists of 4 NPCs designed using Reallusion Character Creator 4 and animated using iClone 8. We use iClone 8′s Acculips function to animate the lip movements of the NPC based on text inputs. We create the 4 NPCs to represent a broad range of age groups, ethnicities, and races. The 4 NPCs consists of one interactive maître d and 3 non-interactive patrons. The maître d NPC interacts with the participants when they are within the simulation. As shown on the left of Fig. 4, we attire the maître d NPC with a white button-up shirt, black pants, and an apron. The 3 non-interactive NPC patrons are shown on the right of Fig. 4. One non-interactive patron NPC is seated at the bar area and does not interact with the other NPCs. We place two non-interactive patron NPCs on a seated are table closest to Standing Table 3. These two non-interactive NPCs engage in typical office talk dialog throughout the simulation.

### 4.5 Sounds

To replicate the verbal and non-verbal sounds that a participant may encounter in a real-world café, we use ElevenLabs’ Multilingual v2 to generate the following sounds:

1. **Verbal Sounds:** When the participant approaches the podium, the maître d’ NPC initiates dialogue by asking “Hello, welcome to Flavor and Vine. Would you like to dine in or take out?” followed by questions one might expect in a public dining setting. In the case of a 3-minute wait time, the maître d’ NPC will first tell the participant “I will be with you shortly.” before initiating the main greeting after the wait time has been completed. We use the voice model “Anna D.” with voice ID “0VPW6YsfGkzxile59Ueo" to generate the speech for the maître d’ NPC.
  We also generate a voice for the seated non-interactive NPC engaged in a dialog to replicate a typical café conversation between co-workers. Using voice model “Cassidy" with voice ID `56AoDkrOh6qfVPDXZ7Pt' we generate the dialog “I hope we see Emily before the holidays, we had such a great time at the beach last time. By the way, how is your deal at the office going, hopefully you can close by next month. I am so hungry I can't wait for the wait staff."
2. **Non-Verbal Sounds:** We generate background sounds consisting of ambient music, utensils and plates clacking, liquid pouring, and distant crowd conversations. The ambient music contains elements of a jazz piano tune with no lyrics.

In Table 9 we provide the timing information of when we use the audio assets in each treatment. In the **Time** column we provide time in a Minute:Second format. We use the term **Absent** to indicate that the audio asset was not used for the particular treatment. We use the term **(variable)** to indicate that the audio event can occur at different times depending on the interactions of the participant. The audio files referenced in Table 9 can be found in the Assets folder in our repository.

**Table 9:** Event times and audio assets for treatments 00, 03, 30, and 33. Link: https://github.com/Terascale-All-sensing-Research-Studio/Dine-In-or-Take-Out-Cafe-Dataset/tree/main/Assets.

Time	Event	Audio Asset Name 00 and 03	Audio Asset Name 30 and 33
0:00	Participant spawned into simulation	TrimmedCafeAmbientNoise.mp3	TrimmedCafeAmbient Noise.mp3
(variable) When participant reaches NPC maître d'	NPC maître d' says: “I will be with you shortly.”	Absent	WithYouShortly.mp3
(variable) When participant reaches NPC maître d'	NPC maître d' says: “Hello, welcome to Flavor and Vine. Would you like to dine in or take out?”	Welcome.mp3	Welcome.mp3
(variable) If participant does not engage with the NPC maître d' after 1-minute of the Welcome.mp3 audio playing	NPC maître d' says: “Would you like to dine in or take out?”	WelcomeLoop.mp3	WelcomeLoop.mp3
(variable) Upon selecting “Dine In”	NPC maître d' says: “Unfortunately, we only have standing tables available right now. Will that work for you?”	WillStandingTableWork.mp3	WillStandingTable Work.mp3
(variable) Upon selecting “Take Out”	NPC maître d' says: “Ok, right this way.”	OkRightThisWay.mp3	OkRightThisWay.mp3
(variable) Upon selecting “Yes Standing Table”	NPC maître d' says: “Ok, right this way.”	OkRightThisWay.mp3	OkRightThisWay.mp3
(variable) Upon selecting “No Standing Table”	NPC maître d' says: “Ok, will take out be ok for you?”	WillTakeoutWork.mp3	WillTakeoutWork.mp3
(variable) Upon selecting “Yes Take Out”	NPC maître d' says: “Ok, right this way.”	OkRightThisWay.mp3	OkRightThisWay.mp3
(variable) Upon selecting “No Take Out”	NPC maître d' says: “Sorry about that.”	Sorry.mp3	Sorry.mp3
(variable) If participant reaches within vicinity of NPC Female40s	NPC Female40s says: “I hope we see Emily before the holidays, we had such a great time at the beach last time. By the way, how is your deal at the office going, hopefully you can close by next month. I am so hungry I can't wait for the wait staff.” in a loop	FemaleBackgroundConvo.mp3	FemaleBackground Convo.mp3

Participants were excluded from our study if any of the following criteria were met:

1. Age 18 or younger on the day of the study. Such participants are considered minors in the United States and would require parental consent.
2. Prisoners and vulnerable populations were excluded due to the additional oversight needed for safe participation.
3. Injuries to fingers that prevent grasping or pressing. While our simulation is controller-free, the movements required to press the virtual buttons may cause complications to individuals with finger injuries.
4. Visual acuity lower than 20/200 with corrective lenses. In the United States, such participants would be considered legally blind and require additional assistance in the real-world setting being studied.
5. History of severe motion sickness in moving vehicles. To reduce the risk of adverse consequences to the participant, we excluded them from our study.

### 4.6 Recruitment method

We recruited participants from the faculty, staff, and students at Wright State University through email and digital flyers posted to the College of Engineering and Computer Science. Wright State University is a predominantly undergraduate institution with approximately 11,000 students located in Dayton, OH, USA. Broader recruitment outside of the College of Engineering and Computer Science was not possible due to institutional protocols on email and campus-wide announcements being sent to other colleges outside of the researchers college, i.e., the College of Engineering and Computer Science. The email and campus flyers included a QR code with a calendar signup page that allowed participants to select available timeslots with the research team.

### 4.7 Demographic data

After confirming that participants did not meet any of the exclusion criteria, each participant was asked to complete the Informed Consent form. After completing the Informed Consent form, we collected participant demographic data. We provided a freeform text box for self-identified gender to ensure that we followed recommended practices from literature for eliciting gender [16]. When collecting ethnicity and race, we used the categorizations provided by the United States National Center for Education Statistics [17]. Given that prior literature suggests that relationships exist between socioeconomic status, isolation, and frustration [18,19], we used education level to measure socioeconomic status [20]. To understand whether participant experience with interactive and immersive environments we asked participants “How often do you play video games?”, “What type of video games do you play?”, “How often do you use a Virtual Reality (VR) device?”, “List the devices you use / have used”, “Do you own a VR device?”, and “List the devices you own”. Finally, to understand participant dining preferences, we asked participants “How frequently do you use take-out services?”, “How frequently do you use dine-in services?”, and “Which of the following do you most often choose?”.

### 4.8 Entry surveys

After completing the demographics form, we ask each participant to complete the Frustration Discomfort Scale (FDS) to understand frustration intolerance, Revised UCLA-20 (R-UCLA) for feelings of loneliness, Social Phobia Inventory (SPIN) to understand the severity of social anxiety, and State Trait Anxiety Inventory (STAI) to measure anxiety levels for the past month.

### 4.9 Study design

After completing the demographics and entry surveys, we randomly assigned participants to either the No wait for maître d’ and No wait for food (coded as 00), No wait for maître d’ and 3-minute wait for food (coded as 03), 3-minutes wait for maître d’ and No wait for food (coded as 30), or 3-minutes wait for maître d’ and 3-minute wait for food (coded as 33) treatment. All 35 of our participants completed the four treatments. Once assigned, the participant was told that they would be spawned into a virtual cafe environment. Participants were informed they would be interacting with a maître d’ using dialogue buttons. We informed participants they could exit the simulation at any time by pressing the Exit button placed on the entrance door. Participants were not informed whether they would be experiencing a delay of interaction with the maître d’ or a delay post menu item selection prior to entering the simulation. Participants were not informed of the length of delay in the immersive environment. We informed participants that if they saw a blue boundary that they were in close proximity to a real-world hazard. A research personnel kept constant watch on the participant to ensure they did not come near any physical hazards. For each participant we recorded: eye gaze hit locations, headset position and orientation, and left and right-hand position and orientation. Once inside the immersive environment, the participant is free to approach the maître d’ podium. The maître d’ will then initiate dialogue based on the treatment case assigned.

### 4.10 Post-treatment survey

After the participant completes each treatment, we re-administered the State Trait Anxiety Inventory (STAI) to understand if there were any changes to their anxiety levels. In addition, we asked participants to answer the following questions:

1. “I felt people in the cafe were: “ with choice on a 5-point Likert scale consisting of Very Close, Somewhat Close, Neither, Somewhat Far, Very Far.
2. “I felt the noise level in the cafe was: “ with choices on a 5-point Likert scale consisting of Very Quiet, Somewhat Quiet, Neither, Somewhat Loud, Very Loud.
3. “What was your frustration level due to the delay?" with choices on a 5-point Likert scale consisting of 1 (Low) to 5 (High).
4. “What was your frustration level due to feelings of isolation?" with choices on a 5-point Likert scale consisting of 1 (Low) to 5 (High).
5. “What was your frustration level due to feeling anxious?" with choices on a 5-point Likert scale consisting of 1 (Low) to 5 (High).
6. “How likely are you to make the same table selection in real life?" with choices on a 5-point Likert scale consisting of Very Likely, Likely, Neither, Unlikely, Very Unlikely.

### 4.11 Exit surveys

We administered the following surveys after the participant finishes all 4 treatments:

1. Frustration Discomfort Scale (FDS): is reapplied to measure if participants report any changes in frustration and discomfort after completing the study.
2. NASA Task Load Index (NASA TLX): to measure perceived workload across mental demand, physical demand, temporal demand, effort, frustration, and performance.
3. System Usability Scale (SUS): to measure the participant’s perception with overall system usability of the VR application.
4. Virtual Reality Sickness Questionnaire (VRSQ): to determine if participants felt any discomfort related to cybersickness during the immersion.

### 4.12 Form validation

We automatically validate the demographics, cybersickness (VRSQ), frustration (FDS), task load (NASA TLX), loneliness (R-UCLA), social phobia (SPIN), anxiety (STAI), usability (SUS), and post treatment forms to ensure that participants complete all entries prior to submission. Our validation checks to make sure that there are no missing form entries prior to submission. As a result, all submitted forms have complete responses and no participant has missing data.

### 4.13 Compensation

We did not compensate participants for taking part in our study.

## Limitations

The dataset was collected on a university campus in the United States with a focus on recruiting participants who were Gen Z or Zillennial adults, thus our participants are college-aged with an average age of 24.83 years (min: 19, max: 53, standard deviation: 5.50). Our participants are predominantly Asian (*N* = 18) or White (*N* = 15), thus reducing the generalizability across all Gen Z or Zillennial individuals. The majority (*N* = 26) of our participants identify as Male and the remainder as Female, which limits the dataset’s ability to generalize across all self-identified gender choices. Our collection was performed in a café setting with a light crowd consisting of three other patrons, thus limiting an understanding of participant behavior in larger crowd densities or other public social settings such as restaurants, bars, movie theaters, convenience stores, or banks. During our collection we used 3-minute waits as opposed to longer duration waits, which may be found in more densely crowded scenarios. Thus, it is likely that participant behavior may change is there are much longer waits. Our study measures loneliness, social phobia, anxiety, and frustration using standard scales as opposed to wearable sensing such as heartrate sensors or galvanic skin resistance sensors. The use of wearables may provide a quantitative metric of how participant response changes during the immersion.

## Ethics Statement

The dataset described in this paper was collected after receiving approval from the Wright State University Institutional Review Board (IRB) under IRB-2025–901. Prior to data collection, all researchers completed the Collaborative Institutional Training Initiative (CITI Program) Human Subjects Research training. The methods described in this paper were performed under the guidelines approved by the IRB.

## CRediT Author Statement

**Elza Ibragimov:** Software, Validation, Investigation, Data Curation, Writing - Review & Editing, Visualization

**Natasha Kholgade Banerjee:** Conceptualization, Methodology, Validation, Resources, Writing - Review & Editing, Supervision

**Sean Banerjee:** Conceptualization, Methodology, Validation, Formal analysis, Resources, Writing - Original Draft, Visualization, Supervision, Project administration

**Ashutosh Shivakumar:** Conceptualization, Methodology, Validation, Resources, Writing - Review & Editing, Supervision, Project administration

## Data Availability

GitHubDine In or Take Out Dataset: User Behavior in an Interactive Virtual Reality Café (Original data)
